# Metabolomic Study on the Therapeutic Effect of the Jianpi Yangzheng Xiaozheng Decoction on Gastric Cancer Treated with Chemotherapy Based on GC-TOFMS Analysis

**DOI:** 10.1155/2021/8832996

**Published:** 2021-03-17

**Authors:** Chao Hou, Hua-Jian Chu, Xiao-Jun Dai, Yin-Qiu Wu, Zheng-Fei He, Yan-Wei Yu, Qing-Yun Lu, Yan-Qing Liu, Xiao-Chun Zhang

**Affiliations:** ^1^College of Medicine, Yangzhou University, Yangzhou 225001, Jiangsu, China; ^2^The State Administration of Traditional Chinese Medicine Key Laboratory of Toxic Pathogens-Based Therapeutic Approaches of Gastric Cancer, Yangzhou University, Yangzhou 225009, Jiangsu, China; ^3^Yizheng Hospital of Traditional Chinese Medicine, Yizheng 211400, Jiangsu, China; ^4^Department of Immunology, Jiangsu University, Zhenjiang 212013, Jiangsu, China; ^5^College of Medicine, Yangzhou Hospital of Traditional Chinese Medicine Affiliated with Nanjing University of Chinese Medicine, Yangzhou 225009, Jiangsu, China

## Abstract

**Objective:**

This study aimed to find new biomarkers of prognosis and metabolomic therapy for gastric carcinoma (GC) treated with chemotherapy and investigate the metabolic mechanism of the Jianpi Yangzheng Xiaozheng (JPYZXZ) decoction in the treatment of GC.

**Methods:**

First, 36 patients with GC were randomly assigned to the treatment (chemotherapy plus JPYZXZ) and control (chemotherapy alone) groups. The clinical efficacy, side effects, and quality of life of patients in the two groups were evaluated after treatment. Then, the serum samples taken from 16 randomly selected patients (eight treatment cases and eight control cases with no evident pattern characters) and eight healthy volunteers were tested to identify the differential metabolite under the gas chromatography-time-of-fight mass spectrometry platform. The relevant metabolic pathways of differential substances were analyzed using multidimensional statistical analysis.

**Results:**

JPYZXZ combined with chemotherapy resulted in a lower risk of leucopenia, thrombocytopenia, and gastrointestinal reaction (*P* < 0.05). Additionally, patients in the treatment group showed a higher Karnofsky (KPS) scale (*P* < 0.05). Compared with healthy persons, patients with GC were found to have 26 significant differential metabolites after chemotherapy; these metabolites are mainly involved in 12 metabolic pathways, such as valine, leucine, and isoleucine biosynthesis. JPYZXZ primarily influences the pentose phosphate pathway; glutathione metabolism; glyoxylate and dicarboxylate metabolism; porphyrin and chlorophyll metabolism; and glycine, serine, and threonine metabolism of patients with GC treated with chemotherapy.

**Conclusions:**

The metabolic characteristics of patients with GC after chemotherapy are mainly various amino acid metabolic defects, especially L-glutamine, L-leucine, L-alloisoleucine, and L-valine. These defects lead to a series of problems, such as decreased tolerance and effectiveness of chemotherapy, increased side effects, decreased immunity, and shortened survival time. In addition, the remarkable upregulation of the gluconolactone level in patients with GC suggests the high proliferative activity of GC cells. Thus, gluconolactone may be used as a potential prognostic and diagnostic evaluation index. Moreover, JPYZXZ can reduce the incidence of ADRs and improve the life quality of patients by the correction of L-glutamine, L-leucine, L-alloisoleucine, and L-valine metabolism deficiency. In addition, gluconolactone metabolism is inhibited by JPYZXZ. Such inhibition may be one of the antitumor mechanisms of JPYZXZ.

## 1. Introduction

Gastric carcinoma (GC), one of the common malignant carcinomas, can easily lead to death once it progresses to an advanced or late stage. Nevertheless, a few interventions can postpone or stop the malignant course of the illness. GC has already become the third most common cause of death as more than one million people worldwide are newly diagnosed with GC every year [1]. Especially in Asia, GC remains to be a critical public health and economic burden [2]. Chemotherapy plays an important role in the treatment of GC, but the curative effect remains limited [3]. In addition, patients usually complain about the adverse drug reactions (ADRs) following chemotherapy. For example, many chemotherapy drugs can lead myelosuppression, severe nausea, and vomiting [4]. These side effects substantially impede the acceptance of therapies and reduce the patients' quality of life (QOL). Therefore, new anticancer drugs with fewer side effects for GC are urgently needed.

As an alternative therapeutic measure, traditional Chinese medicine (TCM) has its unique advantages in enhancing the effect of chemotherapy, increasing drug tolerance, reducing the incidence of ADRs, and improving the immune function and QOL of patients with GC [5, 6]. Jianpi Yangzheng Xiaozheng (JPYZXZ) is a traditional Chinese herbal formulation based on the TCM theory of “Qi invigorating, spleen strengthening, and stasis removing” and has been used in local hospitals for decades. Previous clinical studies have revealed that JPYZXZ considerably improves the QOL of patients, relieves the pain of patients, and prolongs patient survival time for advanced-stage GC in China [7]. Related experiments have also proven that JPYZXZ could inhibit the progression, invasion, epithelial-mesenchymal transition (EMT) and induce the cell apoptosis of GC in vivo and in vitro [8, 9]. Additionally, JPYZXZ could enhance antitumor immunity by regulating the phenotypic change in macrophages from M2 to M1 [10].

Metabolomics, a subdiscipline of biological metabolome technology first propounded by Nicholson, provides a comprehensive and simultaneous analysis about the profile of metabolic changes occurring in living creatures in response to pathophysiological stimuli or genetic modification [11]. Metabolomics has been widely used in various fields such as the mechanism of pharmacodynamics. As it focuses on changes in the overall terminal products, metabolomics is consistent with the overall concept of TCM. In recent years, an increasing number of metabolomic studies related to TCM have been conducted to reveal metabonomics characteristics of different TCM patterns [12] and the mechanism of pharmacodynamics [13–15]. Metabolomics relies principally on nuclear magnetic resonance (NMR) and mass spectra (MS) combined with 1iquid chromatography (LC) or gas chromatography (GC) with precolumn derivatization as a supplementary means to separate the metabolites [16–18]. Gas chromatography-time-of-fight mass spectrometry- (GC-TOFMS-) based metabolomics provides a wide coverage of the central part of cellular metabolism, including glycolysis, citrate cycle, amino acid, and nucleotide metabolism. These pathways are altered in cancer cells and can be targeted by metabolic drugs [19].

The aim of the present study is to explore the effect and action mechanisms of JPYZXZ in metabolic pathways for GC patients treated with chemotherapy. The content is two-fold: (i) to analyze the serum metabolic changes between patients with GC treated with chemotherapy and healthy persons and (ii) to evaluate the effect of JPYZXZ in regulating the serum metabolic profiling of GC treated with chemotherapy based on GC-TOFMS analysis.

## 2. Materials and Methods

### 2.1. Instruments and Reagents

High-purity methoxyamine hydrochloride, fatty acid methyl ester (C7–C30, FAME), anhydrous pyridine (99.5%), and anhydrous sodium sulfate were obtained from Sigma-Aldrich (MO, USA). Derivatization reagents MSTFA (containing 1% TMCS), methanol (Optima LC-MS), and n-hexane were purchased from Thermo Fisher (NJ, USA). Dichloromethane (99.5%), chloroform (99%), and acetone (99.5%) were purchased from China National Pharmaceutical Group Corporation (Beijing, China). Ultrapure water was prepared from a Millipore Reference ultrapure water system (MA, USA) equipped with an LC-MS filter. GC-TOF/MS (LECO Corp, MI, USA) based on silanization-derived GC-TOF/MS was used as an analytical platform for untargeted metabolomics.

### 2.2. Participants

Thirty-six patients with pathological diagnosis of GC at the Yangzhou Hospital of Traditional Chinese Medicine were recruited in this study from April 2018 to December 2018. The Zheng types of GC were identified separately by the unanimous conclusion of three chief physicians on the basis of the TCM criteria of the “Qi deficiency of the spleen and stomach.” The inclusion criteria for GC were as follows: (1) patients with pathological diagnosis of GC, (2) patients that meet the TCM criteria of “Qi deficiency of the spleen and stomach,” (3) KPS scores >60, (4) patients aged 18–75 years old, (5) patients with a life expectancy of at least 6 months, and (6) patients who have provided written informed consent to participate in the study in accordance with the GCP criteria. The exclusion criteria for GC were as follows: (1) patients who cannot swallow oral medications, including digestive tract obstruction and jejunostomy; (2) patients with symptomatic brain metastasis or mental disorder; (3) patients with severe cardiovascular, chronic liver, kidney, blood, endocrine system, or metabolic disease; (4) patients who are pregnant or nursing; and (5) patients who are substance abusers or those with clinical, mental, and social features that interfere the study and informed consent. To avoid the influence of other medications on these small metabolic molecules in the serum, the patients enrolled in this study did not receive any other medications during the treatment with JPYZXZ. All the patients were randomly assigned into two groups: 19 in the treatment group (chemotherapy plus JPYZXZ granules) and 17 in the control group (chemotherapy alone).

Ten age- and gender-matched healthy volunteers without any evidence of tumor were also enrolled during that same period with the following exclusions: (1) patients with severe cardiovascular, chronic liver, kidney, blood, endocrine system, or metabolic disease and (2) patients who are pregnant or nursing. This study was approved by the ethics committee of the Yangzhou Hospital of Traditional Chinese Medicine, and written informed consent was obtained from all the participants.

### 2.3. Preparation of JPYZXZ Decoction

Raw herbs of JPYZXZ decoction were obtained from the Chinese pharmacy of the Yangzhou Hospital of Traditional Chinese Medicine. JYYZXZ includes 15 g of Dangshen, 10 g of Baizhu, 10 g of Fuling, 15 g of Shengyiren, 10 g of Danggui, 15 g of Shanyao, 10 g of Muxiang, 10 g of Baishao, 6 g of Chenpi, 10 g of Baqia, 15 g of Shijianchuan, and 3 g of Zhigancao. All the herbs were mixed and boiled in 500 mL of sterile water for 30 min. The criteria for identifying the quality of the herbs used were in accordance with the 2005 edition of the Chinese Pharmacopoeia (Chinese Pharmacopoeia Commission, Pharmacopoeia of the People's Republic of China, Beijing, People's Medical Publishing House, 2005). Before their use in experiments, the herbs were tested for heavy metals, microbial contamination, and residual pesticides; all results met the safety standards in China.

### 2.4. Drug Administration and Plasma Sample Collection

Patients in the control group were treated with chemotherapy only, and all the chemotherapy regimens refer to the NCCN Guidelines (2019) of GC; details are as follows (any one of them): XELOX, oxaliplatin 130 mg/m^2^ on day 1; capecitabine 1000 mg/m^2^ on days 1–14, twice daily, every 3 weeks; DS-1, docetaxel 40–50 mg/m^2^ on day 1, every 2 weeks; S-1 40–60 mg/m^2^, on days 1–14, twice daily, every 3 weeks; SOX, oxaliplatin 85 mg/m^2^, on day 1, every 2 weeks; S-1 40 mg/m^2^, on days 1–14, twice daily, every 3 weeks. Patients in the treatment group were treated with chemotherapy combined with JPYZXZ decoction. The chemotherapy regimen was the same as that of the control group. One dose of JPYZXZ decoction should be orally administrated every day for at least 6 months. Under the ethical requirements and with the patient's consent, eight blood samples from subjects randomly selected from each group were collected after treatment. Blood samples were recorded and coded as A1, B1, C1, A2, B2, C2, and so on (A, B, and C denote the treatment, control, and healthy groups, respectively; numbers represent the patients in the same group).

### 2.5. Preparation of Serum Samples for Metabolomic Analysis

BD Vacutainer vacuum blood collection tubes were used to collect 7 mL of whole blood from the fasting patients in the morning and kept undisturbed at 4°C for 2 h. The blood was separated and centrifuged (3000 g, 15 min, 4°C) to obtain the serum. The serum was packed in tubes (400 per tube) and stored at −80°C to avoid repeated freezing and thawing and for the sample to remain stable.

### 2.6. GC-TOFMS Analysis

#### 2.6.1. Analysis Conditions

*(1) Chromatography*. Column Rxi-5MS (Restek Corporation, PA, USA) column parameters: 30 m (length) × 0.25 mm (internal diameter), 0.25 (film thickness); oven temperature program 80°C (2 min), 80°C–300°C (12°C/min), 300°C (5.7 min); injection volume (*μ*L); inlet temperature: 270°C; injection mode: no split; carrier gas: helium (99.9999%); carrier gas flow rate (mL/min): 1.0; constant current; transmission line temperature: 270°C.

*(2) Ion Source Type*. EI, detection parameters: electron energy, −70 V; detector voltage, −1400 V; ion source temperature, 220°C; acquisition rate, 25 spectra/s; scan range, 50–500 m/z.

#### 2.6.2. Metabolic Pathway Analysis

Specific metabolites of different patterns obtained by screening were introduced into the online system MetaboAnalyst to analyze the metabolic pathways. Changes in key locations in the network are believed to have a serious effect on the occurrence of events. Therefore, the threshold was set to 0.05 in this study, and the pathways above this threshold were classified as potential metabolic pathways. The metabolic pathways in this study were all generated using KEGG (http://www.genome.jp/kegg/).

#### 2.6.3. Data Processing and Statistical Analysis

State-of-the-art GC-TOFMS technology with fast scan rates can resolve hundreds of metabolite peaks with deconvolution algorithm in a single injection. A typical GC-TOFMS chromatogram of biological samples generates 600–1,000 individual deconvoluted signals. However, molecules containing multiple reactive groups (OH, NH, SH, and NH2) may form multiple (oxime-)TMS derivatives and produce multiple derivatives for the same metabolite. XploreMET (v2.0, Metabo-Profle, Shanghai, China) identifies all of the TMS derivatives using the knowledge of the existing database that was built from the pure chemical standard, calculates the sum of the derivatives, and reports it as one annotated metabolite for the same metabolite. The analysis was performed using SPSS 21.0 statistical software (IBM, NY, USA) in which the measurement data conforming to the normal distribution and the homogeneity of variance were described as mean ± standard deviation. The comparison between multisample groups was analyzed by variance for nonconformity with the normal distribution or variance. The measurement data were described as the median *M* (*Q*1, *Q*3). The Wilcoxon rank-sum test was used for comparison between the two groups. The Kruskal–Wallis H test was used for the multisample comparison of the group design, and the count data were described as the relative number (%). The groups were compared using the *χ*^2^ test. With *α* = 0.05, a *P* value less than 0.05 suggested that the difference was statistically significant.

## 3. Results

### 3.1. Demographic Characteristics and Clinical Effect Data

The demographic characteristics and clinical data of the enrolled subjects are summarized in Table 1. The results demonstrated no significant difference in gender, age, and body mass index (BMI) between the treatment group and control group. Compared with chemotherapy alone, JPYZXZ combined with chemotherapy resulted in a lower risk of leucopenia, thrombocytopenia, and gastrointestinal reaction and showed a higher KPS scale. The demographic characteristics of the enrolled subjects randomly selected from the patients and healthy persons for further metabolomic analysis are summarized in Table 2. The results demonstrated no significant difference in gender, age, and BMI among the three groups.

### 3.2. Visual Inspection of QC/QA

#### 3.2.1. Multivariate Control Chart

**Figure 1:**
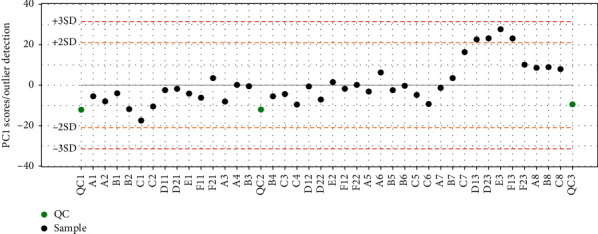
Quality control process: MCC. Each dot represents the summary of global metabolite profiles of each individual. The green dot denotes the pooled QC samples, and the black dots are actual samples. The scores that exceed ±3 SD are generally considered the risk of outliers. The blood samples from the treatment, control, and healthy groups were coded as A, B, and C, respectively.

The multivariate control chart (MCC) is a powerful tool whenever more than one process parameter is used and the effect of multiple parameters are correlated. As shown in Figure 1, each dot in MCC represents each individual subject; the green dots denote the pooled biological QC samples, and the black dots are the actual study samples. In a typical process, most blood samples in our study were within control limits and fluctuated beyond and below the *x*-axis, whereas a few points were near the control limits, and no points were beyond the control limits.

#### 3.2.2. Metabolite Profile Visualization (Intragroup Variations)

**Figure 2:**
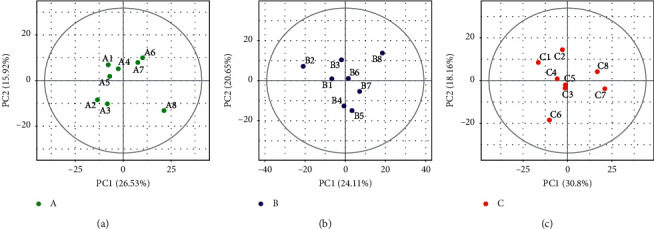
Overview of the metabolic profiles of each subgroup using the PCA score plot.

The initial overview of global metabolic profiles for the subjects from each subgroup, as revealed by the scores plotted with the principal component analysis (PCA) model, was used to provide an informative first look at intragroup variations and detect potential outliers that apparently differ from the others within a group. A value outside the critical limits indicates that the sample is likely to be at risk of outlying observations. As shown in Figure 2, the variation within a group in our blood samples was small.

### 3.3. Metabolites

#### 3.3.1. Metabolite Classes and Compositions Detected in the Samples

**Figure 3:**
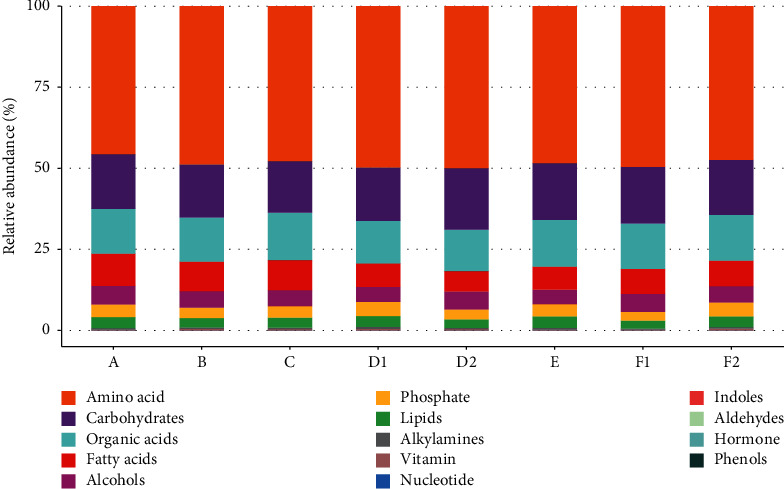
Metabolite classes and compositions detected in the three groups.

Metabolites in the study samples were annotated with the mammalian metabolite database JiaLibTM using a strict matching algorithm incorporated in XploreMET software that uses retention time and fragmentation pattern in the mass spectrum. Metabolite classes and compositions were detected in the samples. The proportional statistics were calculated in accordance with the median percentage of metabolites. As shown in Figure 3, no significant difference was observed in the percentage of metabolites among the three groups.

#### 3.3.2. Differential Metabolite Analysis

3.3.2.1. *Multivariate Data Analysis*

**Figure 4:**
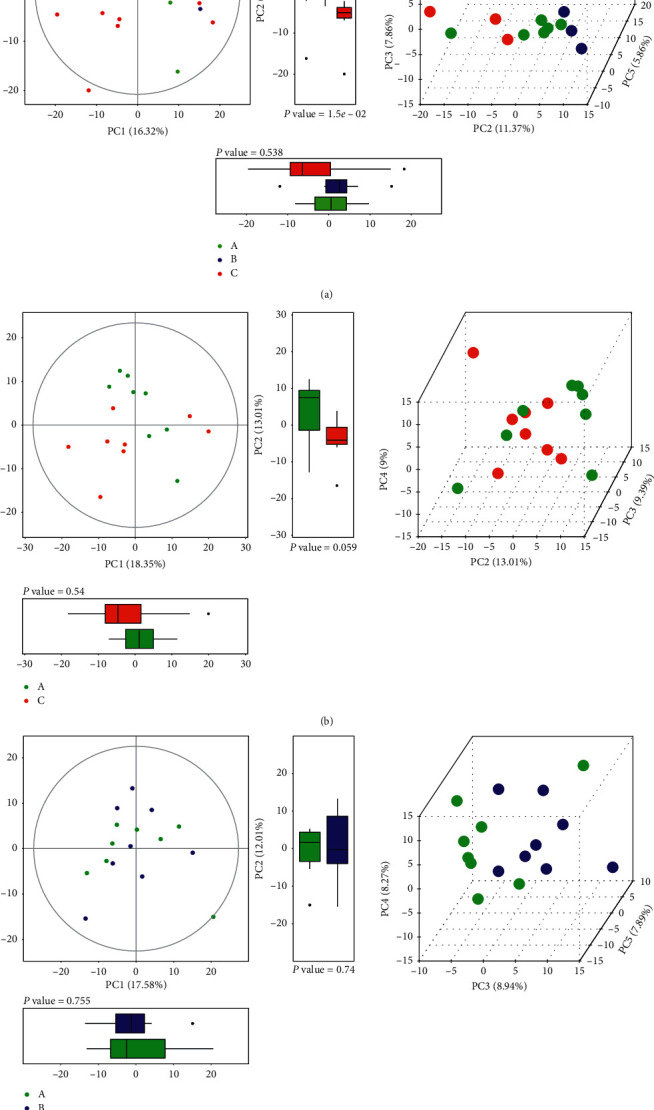
Two- and three-dimensional PCA (score plot and boxplot) (“green plot” A, “blue plot” B, and “red plot” C): (a) PCA of A-B-C. (b) PCA of A-C. (c) PCA of A-B.

**Figure 5:**
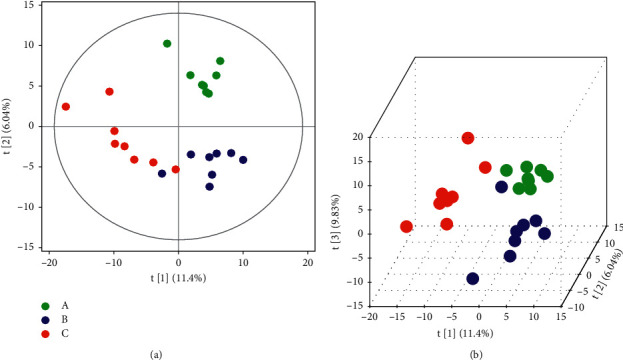
PLS-DA score plot of A-B-C (“green plot” A, “blue plot” B, and “red plot” C): (a) two-dimensional PLS-DA; (b) three-dimensional PLS-DA.

**Figure 6:**
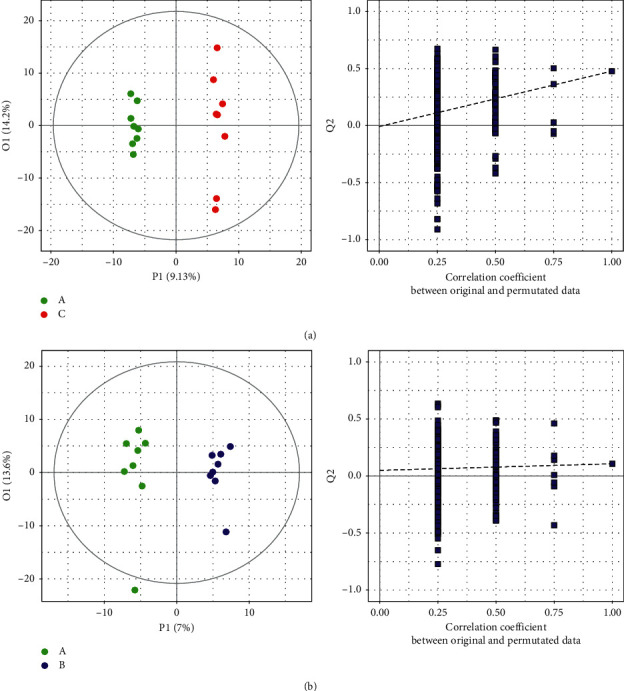
OPLS-DA metabolic map of two groups (“green plot” A, “blue plot” B, and “red plot” C): (a) OPLS-DA metabolic map of A-C. (b) OPLS-DA metabolic map of A-B.

**Figure 7:**
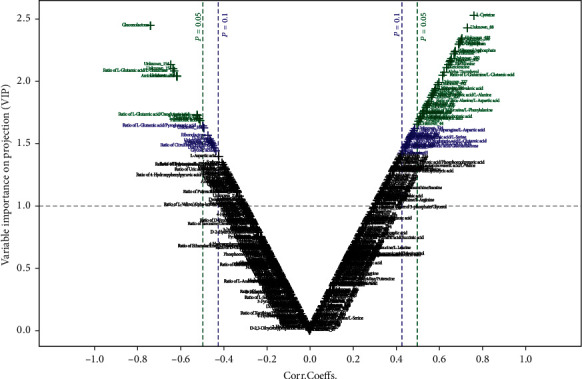
Visualization of differential metabolite profiles using multivariate statistical analysis between groups A and C. The V-plot revealed that the highlighted metabolites from the top right corner are positively correlated with subjects located in the right side of OPLS-DA score plot, indicating that these metabolites are elevated in these subjects as compared with those in the left side of the OPLS-DA score plot, and vice versa. The highlighted metabolites from the top left corner are negatively correlated with subjects located in the right side of the OPLS-DA score plot, indicating that these metabolites are decreased in these subjects as compared with those in the left side of the OPLS-DA score plot.

1. PCA, partial least square discriminant analysis (PLS-DA) and orthogonal partial least square discriminant analysis (OPLS-DA): PCA is an unsupervised recognition model that converts a set of possibly correlated components into a set of linearly uncorrelated variables. Here, PCA was used to determine the difference of the principal components of group A (treatment group), group B (control group), and group C (healthy group). Compared with group A, separation trends were found in groups B and C using two- and three-dimensional PCA analysis (Figure 4). For further validation, PLS-DA and OPLS-DA were used to verify a good separation among the three groups. The results showed that the separation trends became more evident among the three groups (Figures 5 and 6), suggesting that the metabolites in the serum of patients with GC were changed after chemotherapy and JPYZXZ treatment. As a significance threshold 0.4 is generally accepted for Q2Ycum and the intercept of Q2Y with a threshold less than zero indicates a valid model, the results showed that the OPLS-DA model between groups A and C was accepted (Q2Y = 0.477, intercept of Q2 in the *y*-axis is −0.008).
2. Identification of the different metabolites: the OPLS-DA model between groups A and C was valid; thus, we conducted differential metabolite identification-multivariate statistics. The value of variable importance in projection (VIP) score that is greater than 1 is the typical rule for selecting relevant variables. As shown in Figure 7, the result of the volcano plot (V-plot) indicated that the presence of 84 different metabolites (VIP0 > 1) in the serum of patients with GC were changed after chemotherapy.

**Figure 8:**
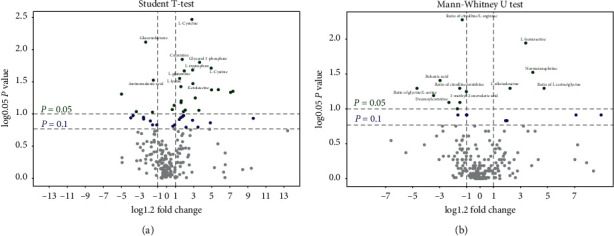
V-plot of differential metabolite profiles. The highlighted metabolites from the top right corner are positively correlated with subjects located in the right side of the OPLS-DA score plot, indicating that these metabolites are increased in these subjects as compared with those in the left side of the OPLS-DA score plot, and vice versa. The highlighted metabolites from the top left corner are negatively correlated with subjects located in the right side of the OPLS-DA plot, indicating that these metabolites are decreased in these subjects as compared with those in the left side of the OPLS-DA score plot. (a) V-plot of differential metabolite profiles between groups A and C. (b) V-plot of differential metabolite profiles between groups A and B.

3.3.2.2 *Univariate Data Analysis.* Univariate data analysis was also used to identify the different metabolites between groups A and C. We found that the concentrations of 205 metabolites differ after chemotherapy (Supplementary Material, Table S2). Moreover, the concentrations of 205 metabolites are disparate after the administration of JPYZXZ (Supplementary Material, Table S3). For further validation in the GC-TOFMS mode, we confirmed 26 significant differential metabolites between groups A and C through GC-TOFMS univariate data analysis (Figure 8(a)). With regard to the comparison between groups A and B, we also determined 13 significant differentiated metabolites (Figure 8(b)).

#### 3.3.3. Identification of the Different Metabolites in the Blood Samples between Groups A and B and Groups A and C

**Figure 9:**
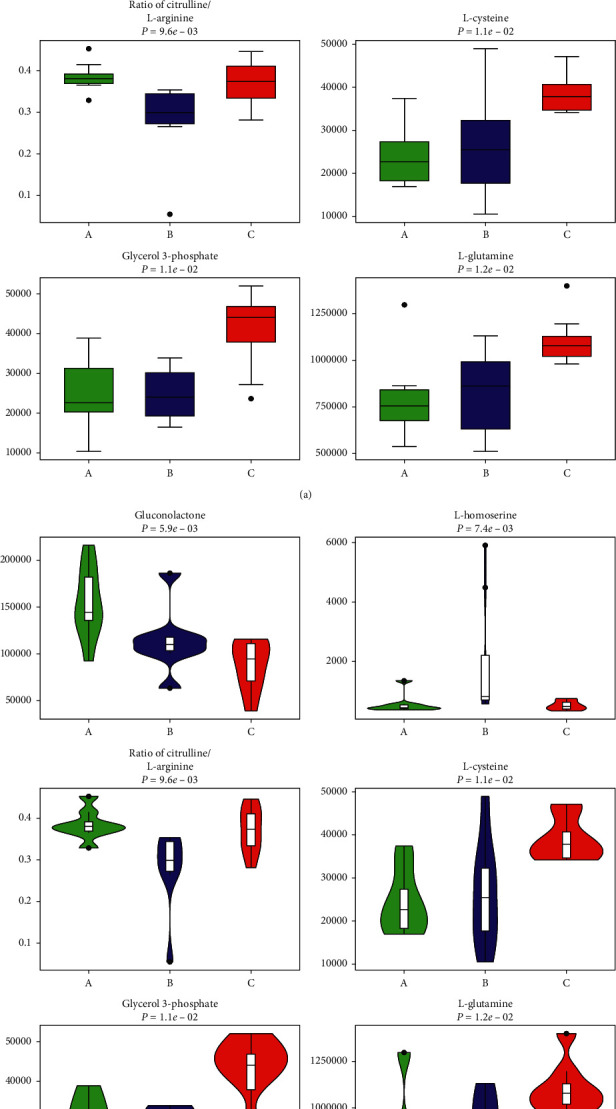
Top-ranked differential metabolites among the three groups (“green boxplots” A, “blue boxplots” B, and “red boxplots” C): (a) box diagram; (b) violin diagram.

**Figure 10:**
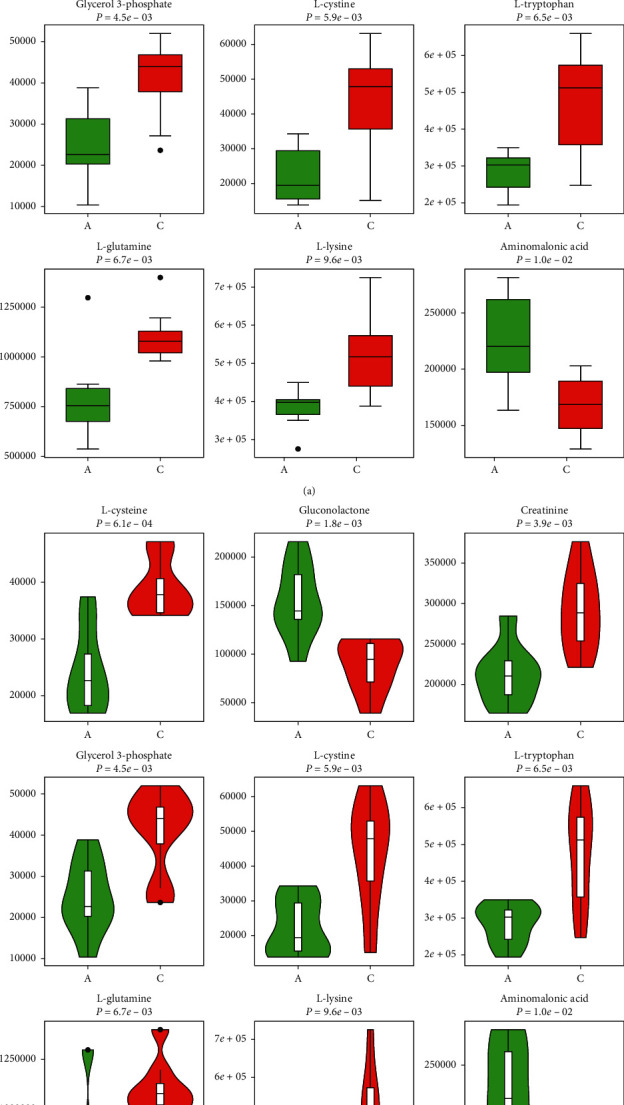
Top-ranked differential metabolites between groups A and C (“green boxplots” A; “red boxplots” C): (a) box diagram; (b) violin diagram.

**Figure 11:**
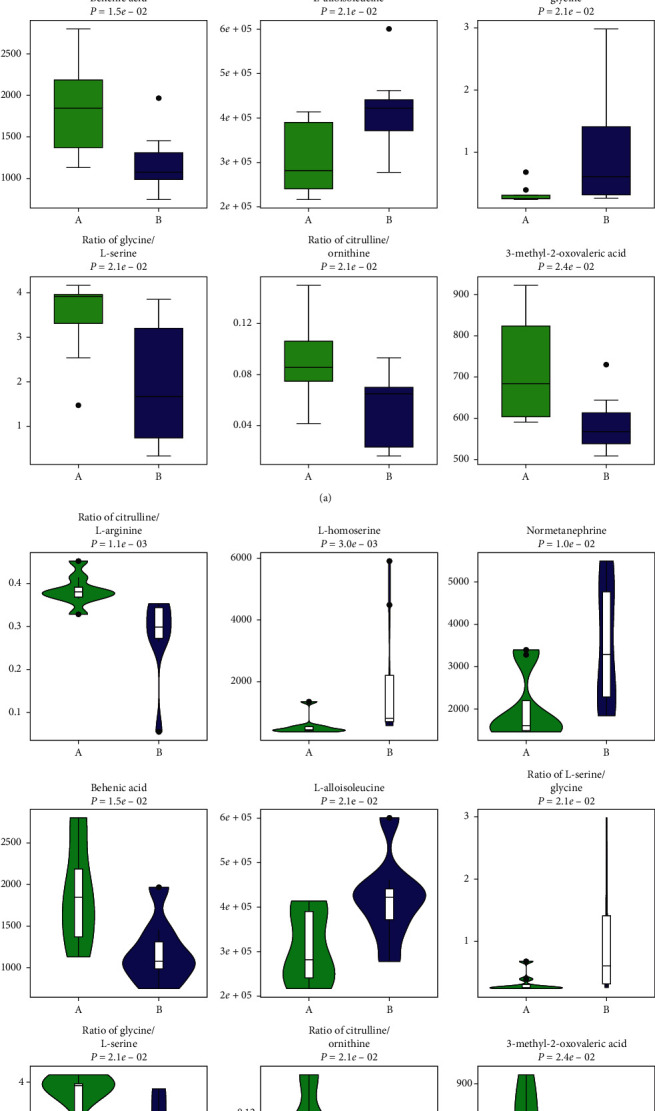
Top-ranked differential metabolites between groups A and B (“green boxplots” A; “red boxplots” B): (a) box diagram; (b) violin diagram.

**Figure 12:**
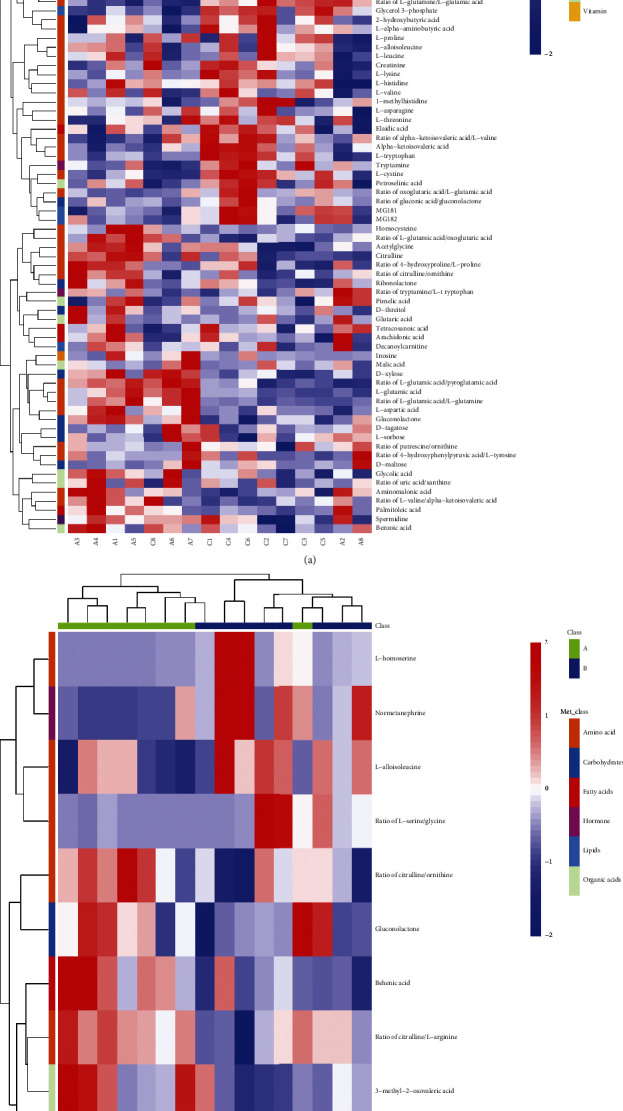
Differential metabolite *Z*-score heat map. Rows: metabolites; Columns: samples. The color key indicates the concentration of metabolites: green suggests downregulation and red suggests upregulation. (a) Groups A and C (b) Groups A and B.

To investigate the relationship between groups of significant different metabolites intuitively, we organized the major metabolites in Table 3. To visualize the change of metabolite, the representative differential metabolites (top ranked) obtained from univariate statistical analysis are illustrated in Figures 9–11. In the present study, cluster analysis of the metabolites with significant different concentrations between groups A and C and groups A and B was conducted to observe their relative variation. The complete dataset was imported to generate a heat map to identify group differences.  Figure 12 shows the relative changes in the concentration of the metabolites in different groups, with the blue band indicating a decreased level of metabolite and the red band indicating an increased level of metabolite. Cluster analysis showed possible links between differential metabolites among three groups.

#### 3.3.4. Metabolic Pathway Enrichment Analysis (MPEA) of Metabolic Pathways

**Figure 13:**
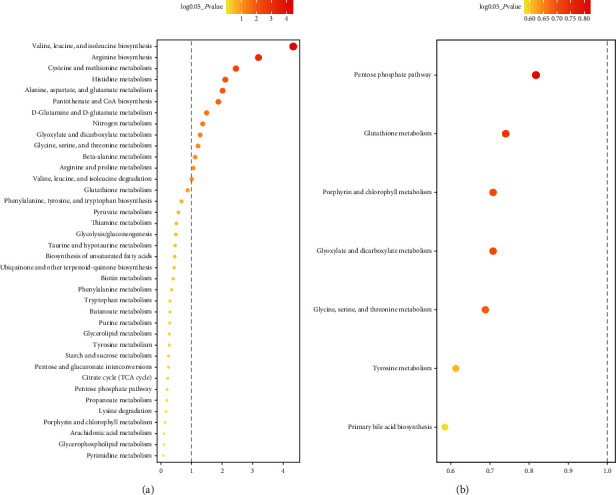
Overview of pathway analysis. The longer the length of the column, the redder the color and the smaller the *P* value, which needs attention. (a) Patients with GC that underwent chemotherapy. (b) Patients with GC that underwent chemotherapy combined with JPYZXZ.

These differentially expressed metabolites were further imported into the online system MetaboAnalyst to analyze the metabolic pathways. Changes in key locations in the network are believed to have a serious effect on the occurrence of events. Therefore, the threshold of key metabolic pathways was set to 0.05 in this study, and the pathways above this threshold were classified as potential metabolic pathways. The MPEA results showed that compared with healthy persons, patients with GC had possessed significantly abnormal amino acid metabolism, such as valine, leucine, and isoleucine biosynthesis and D-glutamine and D-glutamate metabolism (Figure 13(a)), after chemotherapy. The analysis also revealed that JPYZXZ primarily influences the pentose phosphate pathway; glutathione metabolism; glyoxylate and dicarboxylate metabolism; porphyrin and chlorophyll metabolism; and glycine, serine, and threonine metabolism of patients with GC treated with chemotherapy (Figure 13(b)). The upregulated or downregulated metabolites are also provided in Tables 4 and 5, respectively.

## 4. Discussion

### 4.1. Biological Explanation of the Metabolic Pathways Related to Patients with GC after Chemotherapy

Metabolomics is the endpoint of the “omics cascade” and reflects perturbations in metabolites within all biological processes [20]. Given its high sensitivity, metabolomics offers notable advantages in identifying perturbations at an early stage of a disease. Duplicated metabolites have been detected in multiple types of samples in GC and colorectal cancer. In recent years, many metabolomic studies on GC have duplicated detected metabolites in multiple types of samples related to GC, indicating their potential utility as candidate diagnostic biomarkers [21–23]. However, the characteristics of serum metabolomics after chemotherapy for GC have not been reported. One of the objectives of our study is to explore potential metabolic biomarkers of ADRs among patients with GC treated with chemotherapy. The unlimited proliferation of cancer cells requires heavy consumption of amino acids, leading to the body's amino acid metabolism defects. The amino acid metabolism of different cancers, such as breast and colorectal cancer [21, 24], has specificity and guiding significance for the diagnosis, prognosis, and treatment of tumors to some extent [25, 26]. Our data also showed that the different serum metabolites of patients with GC after chemotherapy are mostly amino acids, such as L-cysteine. Other kinds of metabolites, including carbohydrates, such as gluconolactone; lipids, such as glycerol 3-phosphate; vitamins, such as alpha-tocopherol; and organic acids, such as pyruvic acid, also differ significantly (Table 3). The amino acids that cause our concern are L-glutamine, L-leucine, L-alloisoleucine, and L-valine; gluconolactone also caught our attention.

Glutamine, a nonessential amino acid, is consumed by cancer cells at exceedingly high rates to fulfill their energetic and biosynthetic requirements for proliferation. The high rate of glutamine uptake plays a required role in the uptake of essential amino acids and in maintaining the activation of target of rapamycin kinase in cancer cells, especially those driven by KRAS [27, 28]. Moreover, in many cancer cells, glutamine is the primary mitochondrial substrate and is required for the maintenance of mitochondrial membrane potential and integrity and for support of the NADPH production needed for redox control and macromolecular synthesis [28]. Bolzoni [29] found that ASCT2 inhibition in human myeloma cell lines causes a marked decrease in glutamine uptake and a significant fall in cell growth. The metabolism of branched-chain amino acids (BCAAs), including leucine, isoleucine, and valine, are also deregulated in many cancers, specifically affecting the cancer cell state and systemic metabolism in individuals with malignancy [30]. An accumulating body of evidence has demonstrated that BCAAs are essential nutrients for cancer growth and are used by tumors in various biosynthetic pathways as a source of energy. In addition, BCAA metabolic enzymes, such as the cytosolic branched-chain aminotransferase 1 (BCAT1) and mitochondrial branched-chain aminotransferase 2 (BCAT2), have emerged as useful prognostic cancer markers. BCAT1 expression commonly correlates with aggressive cancer growth and progression and has attracted substantial scientific attention in the past few years [31]. The stata of our study revealed that compared with the healthy persons, the level of L-glutamine, L-leucine, L-alloisoleucine, and L-valine declined to varying degrees (Supplementary Material, Tables S1 and S4) in patients with GC after chemotherapy, suggesting that this deficiency in amino acid metabolism is caused by the active proliferation of GC. Related differential metabolic pathways also verified this finding (Table 4). Additionally, malnutrition caused by the downregulation of these amino acids can lead to a series of problems, such as decreased tolerance and effectiveness of chemotherapy, increased side effects, decreased immunity, and shortened survival time [32–34]. Pan found that in a multicenter, cross-sectional study with 2248 cancer patients from 20 hospitals from January to June 2010, patients with gastrointestinal malignancies had a higher rate of undernutrition than other patients. For patients with nutritional risk, the relative risk of adverse events significantly increased with and without nutritional treatment [35]. Therefore, correcting the metabolic defect of these amino acids may benefit the chemotherapy of patients with GC.

As an important branch of glucose metabolism, the pentose phosphate pathway (PPP) is critical for cancer prevention and treatment because NADPH and R5P play important roles in regulating DNA damage response, metabolism, and proliferation in cancer cells. Various enzymes in the PPP have been shown to be potential targets in cancer therapy [36]. Wu [37] found that transcription factor YY1 could promote cell proliferation by directly activating PPP. In our study, a significant upregulation of the gluconolactone level in GC patients treated with chemotherapy was observed, suggesting the high proliferative activity of GC cells.

### 4.2. Antitumor Effect of JPYZXZ

For multicenter clinical validation, JPYZXZ was developed as a “guideline for the diagnosis and treatment of GC by integrative Chinese and Western medicine.” JPYZXZ has been verified to reduce the toxic effects of chemotherapy, improve the activity of macrophage cells, and regulate immune function (Chinese national patent number: zl201410055831.9). Experimental studies have confirmed that JPYZXZ could inhibit the progression, invasion, and EMT of GC; induce the cell apoptosis of GC; and enhance antitumor immunity [8–10]. An HPLC-MS analysis of the JYYZXZ decoction was conducted, and the contents of astilbin, baicalin, hesperidin, glycyrrhizin, paeoniflorin, lobetyolin, adenosine, and costunolide in the decoction were finally determined to be 5.338, 21.86, 60.03, 4.878, 60.68, 3.897, 0.496, and 6.853 *µ*g/g, respectively [10]. Astilbin has been reported to exhibit proapoptotic properties in breast carcinoma cells via modulation of the caspase-dependent pathway, which highlights the feasibility of astilbin as a candidate agent for breast cancer treatment [38]. Baicalin has potential as a therapeutic agent for GC by inducing apoptosis in cancer cells [39]. Glycyrrhizin can induce apoptosis in human stomach cancer KATO III and human promyelotic leukemia HL-60 [40] cells. Paeoniflorin has been revealed to inhibit the migration- and invasion-promoting capacities of GCAFs by targeting microRNA-149 and IL-6. Paeoniflorin is potentially a novel therapeutic agent against cancer microenvironments [41]. He [42] found lobetyolin could induce the apoptosis via the inhibition of ASCT2-mediated glutamine metabolism, which was possibly governed by p53. Meanwhile, costunolide has been proven to induce mitochondria-mediated apoptosis in human gastric adenocarcinoma BGC-823 cells and could be the candidate drug against GC in clinical practice [43].

Commonly used antineoplastic drugs, such as fluorouracil, can induce many inevitable ADRs, mainly including fatigue, anemia, diarrhea, nausea and vomiting, and myelosuppression [44]. By contrast, TCM formulae exhibit low toxicity and high efficiency and multitargets and thus should be used as a priority in combination therapy in treating patients with cancer. In our study, we found that JPYZXZ combined with chemotherapy resulted in a lower risk of leucopenia, thrombocytopenia, and gastrointestinal reaction. Additionally, patients in the treatment group showed higher KPS scale, suggesting that JPYZXZ could reduce the incidence of ADRs, and improved the QOL of patients with GC. Moreover, the serum samples from the treatment, control, and healthy groups were analyzed by GC-TOFMS analysis to reveal the significantly different metabolite among the three groups and evaluate the protective effect and relative mechanism of JPYZXZ. The results of our study indicated that JPYZXZ increases the level of L-glutamine, L-alloisoleucine, and L-leucine to some extent for GC patients treated with chemotherapy (Table 4). However, the level of gluconolactone decreased significantly after the treatment of JPYZXZ. Enrichment analysis showed that PPD is the most different metabolic pathway between the treatment and control groups (Figure 13(b)). Our data suggest that JPYZXZ could reduce the incidence of ADRs and improve the QOL of patients via the correction of tumor-related amino acid metabolism deficiency and inhibition of gluconolactone metabolism. However, the specific mechanisms of the metabolomic effect of JPYZXZ on patients with GC treated with chemotherapy must be further elaborated.

## 5. Conclusion

In this study, we first confirmed the antitumor clinical efficacy of JPYZXZ. Then, the serum metabolic changes in GC patients treated with chemotherapy were analyzed. Lastly, our study preliminarily revealed the anticancer mechanism of JPYZXZ in metabolomics. The metabolic characteristics of patients with GC after chemotherapy are mainly various amino acid metabolic defects, especially L-glutamine, L-leucine, L-alloisoleucine, and L-valine, which lead to a series of problems, such as decreased tolerance and effectiveness of chemotherapy, increased side effects, decreased immunity, and shortened survival time. In addition, a remarkable upregulation of the gluconolactone level in the patients with GC suggests the high proliferative activity of GC cells. Thus, gluconolactone may be used as a potential prognostic and diagnostic evaluation index. JPYZXZ could reduce the incidence of ADRs and improve the QOL of patients via the correction of L-glutamine, L-leucine, L-alloisoleucine, and L-valine metabolism deficiency. In addition, gluconolactone metabolism was also inhibited by JPYZXZ, which may be one of the antitumor mechanisms of JPYZXZ.

## Figures and Tables

**Table 1:** The demographic characteristics and clinical effect data of subjects.

Characteristics	Treatment group (*n* = 19)	Control group (*n* = 17)	*P* value
Gender (male/female)	11/8	10/7	0.955
Age (years)	59.26 ± 6.89	59.78 ± 4.81	0.797
BMI (kg/m^2^)	19.73 ± 2.23	19.62 ± 2.00	0.878
Leucopenia (events/total)	6/19	11/17	0.049 ^*∗*^
Thrombocytopenia (events/total)	2/19	7/19	0.041 ^*∗*^
Liver dysfunction (events/total)	3/19	4/17	0.434
Renal dysfunction (events/total)	2/19	3/17	0.445
Neurotoxicity (events/total)	2/19	4/17	0.276
Gastrointestinal reaction (events/total)	3/19	8/17	0.047 ^*∗*^
Karnofsky performance status (KPS)	78.94 ± 6.34	73.32 ± 5.98	0.011 ^*∗*^
Disease control rate (%)	15.8	17.6	0.599

^*∗*^*P* < 0.05, compared with the control group.

**Table 2:** The demographic characteristics of subjects for further metabolomic analysis.

Characteristics	Treatment group	Control group	Health group	*P* value
Gender (male/female)	5/3	5/3	4/4	0.842
Age (years)	62.14 ± 5.55	60.50 ± 4.63	60.50 ± 3.78	0.610
BMI (kg/m^2^)	18.83 ± 2.45	20.70 ± 1.93	20.71 ± 1.34	0.111

**Table 3:** Different metabolites between two groups.

No.	Class	Name	C vs A				B vs A		
			VIP^a^	FC^b^	*P* ^c^	Tendency^d^	FC	*P*	Tendency
1	Amino acid	L-Cysteine	2.5	1.667	<0.001	↑^#^	1.123	0.959	↑
2	Carbohydrates	Gluconolactone	2.4	0.656	0.0018	↓^#^	0.761	0.038	↓ ^*∗*^
3	Amino acid	Creatinine	2.2	1.371	0.0039	↑^#^	0.946	0.878	↓
4	Lipids	Glycerol 3-phosphate	2.2	1.946	0.0045	↑^#^	1.603	0.959	↑
5	Amino acid	L-Cystine	2.3	2.461	0.0059	↑^#^	1.307	0.279	↑
6	Amino acid	L-Tryptophan	2.3	1.692	0.0065	↑^#^	0.923	0.959	↓
7	Amino acid	L-Glutamine	2.1	1.429	0.0067	↑^#^	1.141	0.721	↑
8	Amino acid	L-Lysine	2.2	1.301	0.0096	↑^#^	0.992	0.959	↓
9	Amino acid	Aminomalonic acid	2	0.765	0.01	↓ ^*∗*^	1.075	0.721	↑
10	Amino acid	Ketoleucine	2.1	1.701	0.012	↑ ^*∗*^	0.708	0.382	↓
11	Amino acid	L-Methionine	2.1	1.335	0.014	↑ ^*∗*^	1.074	0.959	↑
12	Vitamin	Alpha-Tocopherol	2.1	2.843	0.016	↑ ^*∗*^	0.916	0.959	↓
13	Amino acid	Ratio of L-glutamine/L-glutamic acid	2.0	2.478	0.016	↑ ^*∗*^	1.497	0.878	↑
14	Organic acids	Pyruvic acid	1.9	3.82	0.017	↑ ^*∗*^	0.949	0.878	↓
15	Amino acid	Ratio of pyruvic acid/L-alanine	1.9	3.643	0.018	↑ ^*∗*^	0.995	1.000	↓
16	Amino acid	Ratio of L-glutamic acid/L-glutamine	2.1	0.402	0.020	↓ ^*∗*^	0.693	0.878	↓
17	Amino acid	Alpha-Ketoisovaleric acid	1.9	1.785	0.024	↑ ^*∗*^	0.901	0.878	↓
18	Amino acid	Ratio of L-tyrosine/L-phenylalanine	1.8	1.343	0.027	↑ ^*∗*^	0.965	0.721	↓
19	Amino acid	Ratio of beta-alanine/L-aspartic acid	1.8	1.339	0.029	↑ ^*∗*^	1.37	0.382	↑
20	Amino acid	L-Tyrosine	1.7	1.165	0.033	↑ ^*∗*^	0.785	0.798	↓
21	Amino acid	L-Alpha-aminobutyric acid	1.7	1.121	0.041	↑ ^*∗*^	0.736	0.442	↓
22	Amino acid	2-Hydroxybutyric acid	1.7	1.459	0.042	↑ ^*∗*^	1.08	1.000	↑
23	Lipids	MG181	1.7	1.92	0.042	↑ ^*∗*^	0.948	1.000	↑
24	Amino acid	L-Leucine	1.7	1.406	0.045	↑ ^*∗*^	1.439	0.083	↑
25	Amino acid	Ratio of L-glutamic acid/oxoglutaric acid	1.7	0.543	0.045	↓ ^*∗*^	1.031	1.000	↑
26	Amino acid	Acetyl glycine	1.7	0.752	0.046	↓ ^*∗*^	1.073	0.721	↑
27	Amino acid	L-Alloisoleucine	1.7	—	0.042	↑ ^*∗*^	1.5	0.021	↑ ^*∗*^
28	Amino acid	L-Valine	1.6	1.293	0.064	↑	1.222	0.328	↑
29	Amino acid	Ratio of citrulline/L-arginine	—	0.981	0.584	↓	0.786	0.0011	↓^#^
30	Amino acid	L-Homoserine	—	1.096	0.739	↑	1.853	0.003	↑^#^
31	Hormone	Normetanephrine	—	1.185	0.603	↑	2.038	0.010	↑ ^*∗*^
32	Fatty acids	Behenic acid		0.776	0.293	↓	0.582	0.015	↓ ^*∗*^
34	Amino acid	Ratio of L-serine/glycine	—	1.117	0.842	↑	2.374	0.021	↑ ^*∗*^
35	Amino acid	Ratio of glycine/L-serine		0.895	0.970	↓	0.426	0.021	↓ ^*∗*^
36	Amino acid	Ratio of citrulline/ornithine	1.5	0.82	0.083	↓	0.76	0.021	↓ ^*∗*^
37	Organic acids	3-Methyl-2-oxovaleric acid	—	1.091	0.410	↑	0.83	0.024	↓ ^*∗*^
38	Lipids	Decanoylcarnitine	1	0.627	0.200	↓	0.534	0.028	↓ ^*∗*^
39	Amino acid	Glycine	—	1.082	0.920	↑	0.656	0.038	↓ ^*∗*^
40	Amino acid	Ratio of ketoleucine/L-leucine	—	1.249	0.471	↑	0.736	0.050	↓

^a^VIP values were obtained from cross-validated OPLS-DA models with a threshold of 1. ^b^Fold change was calculated as the ratio of the mean metabolite levels between two groups. ^c^*P* values were calculated from two-tailed student's T-test or Mann–Whitney *U* test or with a threshold of 0.05 and 0.01. ^d^↑, content increased; ↓, content decreased; ^#^Compared with group A, *P* < 0.01;  ^*∗*^Compared with group A, *P* < 0.05.

**Table 4:** Differential metabolic pathway between groups A and C.

Pathway name	*P*	Impact	Down^#^	Up^#^
Valine, leucine, and isoleucine biosynthesis	<0.001	0.75	Alpha-Ketoisovaleric acid; ketoleucine; L-leucine; L-threonine; L-valine	—
Arginine biosynthesis	<0.001	0.31	L-Glutamine; urea	Citrulline; L-aspartic acid; L-glutamic acid
Cysteine and methionine metabolism	<0.001	0.24	L-Alpha-aminobutyric acid; L-cysteine; L-cystine; L-methionine; pyruvic acid	Homocysteine
Histidine metabolism	0.00183	0.33	1-Methylhistidine; L-histidine	L-Aspartic acid; L-glutamic acid
Alanine, aspartate, and glutamate metabolism	0.00283	0.48	L-Glutamine; pyruvic acid	L-Asparagine; L-aspartic acid; L-glutamic acid
Pantothenate and CoA biosynthesis	0.0036	0.17	Alpha-Ketoisovaleric acid; L-cysteine; L-valine	L-Aspartic acid
D-Glutamine and D-glutamate metabolism	10.0114	0.67	L-Glutamine	L-Glutamic acid
Nitrogen metabolism	0.0167	0.25	L-Glutamine	L-Glutamic acid
Glyoxylate and dicarboxylate metabolism	0.0214	0.08	L-Glutamine; pyruvic acid	Glycolic acid; L-glutamic acid
Glycine, serine, and threonine metabolism	0.0264	0.05	Dimethylglycine; L-cysteine; L-threonine; pyruvic acid	—
Beta-Alanine metabolism	0.0351	0.05	L-Histidine	L-Aspartic acid; spermidine
Arginine and proline metabolism	0.0418	0.10	L-Proline; pyruvic acid	L-Glutamic acid; spermidine
Valine, leucine, and isoleucine degradation	0.0492	0.08	Alpha-Ketoisovaleric acid; ketoleucine; L-leucine; L-valine	—

^#^Compared with group C.

**Table 5:** Differential metabolic pathway between groups A and B.

Pathway name	*P*	Impact	Up^#^	Down^#^
Pentose phosphate pathway	0.0864	0.03	—	Gluconolactone
Glutathione metabolism	1.09	0.03	—	Glycine
Glyoxylate and dicarboxylate metabolism	1.20	0.08	—	Glycine
Porphyrin and chlorophyll metabolism	1.20	0.04	—	Glycine
Glycine, serine, and threonine metabolism	1.27	0.21	—	Glycine

^#^Compared with group A.

## Data Availability

Data were detected in April 2019. The data used to support the findings of this study are available from the corresponding author upon request.

## Supplementary Materials

Supplementary Materials Table S1: different metabolites via multivariate statistical analysis (VIP > 1, group C vs group A); Table S2: different metabolites via univariate statistical analysis (group C vs group A); Table S3: different metabolites via univariate statistical analysis (group B vs group A).
