# Supplementary material for: Metabolomic Study on the Therapeutic Effect of the Jianpi Yangzheng Xiaozheng Decoction on Gastric Cancer Treated with Chemotherapy Based on GC-TOFMS Analysis
Source: Evid Based Complement Alternat Med. 2021 Mar 17;2021:8832996. doi: 10.1155/2021/8832996 (PMC7994103; doi:10.1155/2021/8832996)
Supplement: Supplementary Materials — Table S1: different metabolites via multivariate statistical analysis (VIP > 1, group C vs group A); Table S2: different metabolites via univariate statistical analysis (group C vs group A); Table S3: different metabolites via univariate statistical analysis (group B vs group A). [file 8832996.f1.zip › 8832996.f1/Supplementary material 1.pdf]

Class	Name	VIP	Corr.Coef	p
Alkylamir	Hydroxyla	1.5	0.45	0.083
Alkylamir	Spermidir	1.2	-0.38	0.151
Amino Aci	L-Cysteir	2.5	0.76	6.00E-04
Amino Aci	L-Cystine	2.3	0.7	2.60E-03
Amino Aci	L-Glutami	2.3	0.69	2.90E-03
Amino Aci	L-Tryptop	2.3	0.69	3.00E-03
Amino Aci	Creatinir	2.2	0.67	4.60E-03
Amino Aci	L-Lysine	2.2	0.65	6.40E-03
Amino Aci	L-Methior	2.1	0.64	7.20E-03
Amino Aci	Ketoleuci	2.1	0.64	8.10E-03
Amino Aci	Ratio of	2.1	-0.63	9.20E-03
Amino Aci	Ratio of	2	0.62	1.10E-02
Amino Aci	Aminomalc	2	-0.61	1.10E-02
Amino Aci	Alpha-ket	1.9	0.58	1.80E-02
Amino Aci	Ratio of	1.9	0.57	2.20E-02
Amino Aci	Ratio of	1.8	0.55	2.60E-02
Amino Aci	Ratio of	1.8	0.53	3.40E-02
Amino Aci	Methylcys	1.7	0.53	3.70E-02
Amino Aci	L-Leucine	1.7	0.52	3.70E-02
Amino Aci	Ratio of	1.7	-0.52	3.80E-02
Amino Aci	Acetylgly	1.7	-0.52	3.90E-02
Amino Aci	L-Alpha-a	1.7	0.52	4.10E-02
Amino Aci	L-Alloisc	1.7	0.51	4.20E-02
Amino Aci	L-Glutami	1.7	-0.51	4.30E-02
Amino Aci	2-Hydroxy	1.7	0.51	4.30E-02
Amino Aci	L-Tyrosir	1.7	0.51	4.60E-02
Amino Aci	Ratio of	1.6	-0.49	0.051
Amino Aci	Ratio of	1.6	0.48	0.058
Amino Aci	L-Valine	1.6	0.47	0.064
Amino Aci	Ratio of	1.5	0.47	0.069
Amino Aci	Dimethylg	1.5	0.47	0.069
Amino Aci	Ratio of	1.5	0.46	0.075
Amino Aci	Ratio of	1.5	-0.45	0.083
Amino Aci	L-Histidi	1.5	0.44	0.087
Amino Aci	Urea	1.4	0.43	0.093
Amino Aci	L-Proline	1.4	0.42	0.101
Amino Aci	L-Asparti	1.4	-0.42	0.104
Amino Aci	Ratio of	1.3	-0.4	0.124
Amino Aci	Ratio of	1.3	0.4	0.128
Amino Aci	1-Methylh	1.3	0.39	0.136
Amino Aci	Ratio of	1.3	0.39	0.137
Amino Aci	Ratio of	1.2	-0.37	0.153
Amino Aci	Homocyste	1.2	-0.37	0.163
Amino Aci	Citrullir	1.2	-0.36	0.165
Amino Aci	L-Asparag	1.1	0.35	0.19
Amino Aci	Ratio of	1.1	-0.33	0.206
Amino Aci	L-Threoni	1.1	0.32	0.228
Amino Aci	Ratio of	1	0.31	0.236
Amino Aci	Ratio of	1	-0.3	0.254
Carbohydr	Gluconola	2.4	-0.74	1.10E-03

Carbohydr	Ribonolac	1.6	-0.47	0.065
Carbohydr	D-Xylose	1.5	-0.46	0.071
Carbohydr	Ratio of	1.5	0.44	0.084
Carbohydr	L-Sorbose	1.3	-0.41	0.119
Carbohydr	1,5-Anhyd	1.3	0.39	0.131
Carbohydr	D-Tagatos	1.1	-0.34	0.201
Carbohydr	D-Threito	1.1	-0.34	0.201
Carbohydr	D-Maltose	1	-0.3	0.252
Fatty Aci	Palmitole	1.7	-0.51	4.50E-02
Fatty Aci	Arachidon	1.2	-0.36	0.172
Fatty Aci	Elaidic a	1.1	0.33	0.211
Fatty Aci	Tetracosa	1	-0.31	0.249
Indoles	Tryptamin	1.4	0.43	0.098
Indoles	Ratio of	1.3	-0.4	0.124
Indoles	3-Indolep	1.1	0.32	0.222
Lipids	Glycerol	2.2	0.68	4.00E-03
Lipids	MG181	1.7	0.53	3.70E-02
Lipids	Decanoylc	1	-0.31	0.235
Lipids	MG182	1	0.31	0.251
Nucleotid	Inosine	1.1	-0.34	0.192
Nucleotid	Ratio of	1.1	0.34	0.193
Organic A	Pyruvic a	1.9	0.58	1.90E-02
Organic A	L-Lactic	1.6	0.48	0.062
Organic A	Ratio of	1.5	0.45	0.08
Organic A	Glycolic	1.4	-0.43	0.093
Organic A	Ratio of	1.3	0.41	0.12
Organic A	Ratio of	1.3	-0.39	0.137
Organic A	2-Hydroxy	1.3	0.38	0.142
Organic A	Glutaric	1.3	-0.38	0.144
Organic A	Pimelic a	1.2	-0.35	0.18
Organic A	Benzoic a	1.1	-0.34	0.199
Organic A	Petroseli	1.1	0.32	0.219
Organic A	Malic aci	1.1	-0.32	0.231
Vitamin	Alpha-Toc	2.1	0.62	9.70E-03
