# Supplementary material for: Metabolomic Study on the Therapeutic Effect of the Jianpi Yangzheng Xiaozheng Decoction on Gastric Cancer Treated with Chemotherapy Based on GC-TOFMS Analysis
Source: Evid Based Complement Alternat Med. 2021 Mar 17;2021:8832996. doi: 10.1155/2021/8832996 (PMC7994103; doi:10.1155/2021/8832996)
Supplement: Supplementary Materials — Table S1: different metabolites via multivariate statistical analysis (VIP > 1, group C vs group A); Table S2: different metabolites via univariate statistical analysis (group C vs group A); Table S3: different metabolites via univariate statistical analysis (group B vs group A). [file 8832996.f1.zip › 8832996.f1/Supplementary material 2.pdf]

Class	Name	Pvalue	FC
Amino Acid	L-Cysteine	6.10E-04	1.667
Carbohydrates	Gluconolactone	1.80E-03	0.656
Amino Acid	Creatinine	3.90E-03	1.371
Lipids	Glycerol 3-phosphate	4.50E-03	1.946
Amino Acid	L-Cystine	5.90E-03	2.461
Amino Acid	L-Tryptophan	6.50E-03	1.692
Amino Acid	L-Glutamine	6.70E-03	1.429
Amino Acid	L-Lysine	9.60E-03	1.301
Amino Acid	Aminomalonic acid	1.00E-02	0.765
Amino Acid	Ketoleucine	1.20E-02	1.701
Amino Acid	L-Methionine	1.40E-02	1.335
Vitamin	Alpha-Tocopherol	1.60E-02	2.843
Amino Acid	Ratio of L-Glutamine/L-Glutamic acid	1.60E-02	2.478
Organic Acids	Pyruvic acid	1.70E-02	3.82
Amino Acid	Ratio of Pyruvic acid/L-Alanine	1.80E-02	3.643
Amino Acid	Ratio of L-Glutamic acid/L-Glutamine	2.00E-02	0.402
Amino Acid	Alpha-ketoisovaleric acid	2.40E-02	1.785
Amino Acid	Ratio of L-Tyrosine/L-Phenylalanine	2.70E-02	1.343
Amino Acid	Ratio of Beta-Alanine/L-Aspartic acid	2.90E-02	1.339
Amino Acid	L-Tyrosine	3.30E-02	1.165
Amino Acid	L-Alpha-aminobutyric acid	4.10E-02	1.121
Amino Acid	2-Hydroxybutyric acid	4.20E-02	1.459
Lipids	MG181	4.20E-02	1.92
Amino Acid	L-Leucine	4.50E-02	1.406
Amino Acid	Ratio of L-Glutamic acid/Oxoglutaric acid	4.50E-02	0.543
Amino Acid	Acetylglycine	4.60E-02	0.752
Organic Acids	L-Lactic acid	0.054	1.411
Fatty Acids	Palmitoleic acid	0.054	0.508
Amino Acid	Methylcysteine	0.056	1.386
Amino Acid	L-Glutamic acid	0.058	0.627
Amino Acid	L-Alloisoleucine	0.059	1.333
Carbohydrates	Ribonolactone	0.06	0.485
Amino Acid	Ratio of Pyruvic acid/L-Serine	0.061	5.757
Amino Acid	Ratio of L-Glutamic acid/Pyroglutamic acid	0.063	0.63
Amino Acid	L-Valine	0.064	1.293
Amino Acid	Dimethylglycine	0.067	1.69
Carbohydrates	D-Xylose	0.069	0.72
Organic Acids	Ratio of Pyruvic acid/L-Lactic acid	0.075	2.42
Organic Acids	Glycolic acid	0.083	0.82
Amino Acid	Ratio of Citrulline/Ornithine	0.083	0.754
Alkylamines	Hydroxylamine	0.083	1.177
Amino Acid	Urea	0.088	1.135
Amino Acid	Ratio of Oxoglutaric acid/L-Glutamic acid	0.092	1.885
Amino Acid	Ratio of L-Asparagine/L-Aspartic acid	0.093	1.481
Amino Acid	L-Histidine	0.101	1.175
Carbohydrates	Ratio of Gluconic acid/Gluconolactone	0.102	2.381
Carbohydrates	1,5-Anhydrosorbitol	0.105	1.626
Carbohydrates	L-Sorbose	0.109	0.824
Organic Acids	Ratio of Pyruvic acid/Phosphoenolpyruvic aci[illegible]	0.109	11.52
Organic Acids	Glutaric acid	0.112	0.785

Indoles	Tryptamine	0.113	1.267
Amino Acid	Ratio of 4-Hydroxyproline/L-Proline	0.117	0.561
Organic Acids	Ratio of Uric acid/Xanthine	0.125	0.603
Amino Acid	L-Proline	0.125	1.389
Indoles	Ratio of Tryptamine/L-Tryptophan	0.128	0.777
Amino Acid	L-Aspartic acid	0.13	0.787
Amino Acid	Ratio of Urea/L-Arginine	0.136	1.361
Amino Acid	Ratio of Alpha-ketoisovaleric acid/L-Valine	0.136	1.606
Amino Acid	1-Methylhistidine	0.168	1.246
Fatty Acids	Arachidonic acid	0.169	0.776
Alkylamines	Spermidine	0.177	0.915
Amino Acid	Citrulline	0.18	0.672
Organic Acids	2-Hydroxy-3-methylbutyric acid	0.181	1.38
Carbohydrates	D-Threitol	0.184	0.782
Organic Acids	Pimelic acid	0.187	0.737
Amino Acid	Homocysteine	0.188	0.852
Amino Acid	Ratio of 4-Hydroxyphenylpyruvic acid/L-Tyrosi	0.199	0.555
Lipids	Decanoylcarnitine	0.2	0.627
Nucleotide	Ratio of Hypoxanthine/Inosine	0.201	2.816
Carbohydrates	D-Tagatose	0.207	0.907
Organic Acids	Benzoic acid	0.21	0.875
Amino Acid	Ratio of Putrescine/Ornithine	0.214	0.984
Indoles	3-Indolepropionic acid	0.221	1.331
Organic Acids	Malic acid	0.231	0.734
Nucleotide	Inosine	0.236	0.589
Fatty Acids	Elaidic acid	0.244	1.469
Lipids	Ratio of Glycerol 3-phosphate/Glycerol	0.248	1.57
Amino Acid	L-Threonine	0.249	1.39
Amino Acid	L-Asparagine	0.253	0.999
Amino Acid	Ratio of Ornithine/L-Arginine	0.254	1.285
Organic Acids	Petroselinic acid	0.256	1.339
Fatty Acids	Tetracosanoic acid	0.267	0.882
Amino Acid	Ratio of L-Valine/Alpha-ketoisovaleric acid	0.273	0.621
Amino Acid	4-Hydroxyproline	0.275	0.929
Organic Acids	4-Hydroxybenzoic acid	0.276	1.217
Amino Acid	L-Arginine	0.289	0.819
Fatty Acids	Behenic acid	0.293	0.776
Carbohydrates	Erythritol	0.296	0.921
Lipids	MG182	0.305	1.258
Carbohydrates	D-Maltose	0.306	1.047
Carbohydrates	Sucrose	0.307	3.262
Phosphate	Phosphate	0.309	1.042
Carbohydrates	D-Mannose	0.314	1.301
Carbohydrates	Threonic acid	0.321	0.907
Carbohydrates	Alpha-Lactose	0.324	0.738
Amino Acid	L-Alanine	0.326	1.14
NA	3-hydroxypyridine	0.331	0.716
Organic Acids	Pyrrole-2-carboxylic acid	0.332	1.081
Carbohydrates	Allose	0.336	1.561
Amino Acid	Ratio of Sarcosine/Dimethylglycine	0.348	0.67
Carbohydrates	L-Arabitol	0.35	0.976

Organic Acids	Malonic acid	0.355	0.982
Carbohydrates	Gluconic acid	0.364	1.248
Amino Acid	Creatine	0.37	1.536
Carbohydrates	Ratio of D-Fructose/Sucrose	0.381	0.407
Amino Acid	Pyroglutamic acid	0.391	0.971
Carbohydrates	Ribitol	0.393	0.911
Lipids	Cholesterol	0.404	0.994
Nucleotide	Adenine	0.41	0.655
Organic Acids	3-Methyl-2-oxovaleric acid	0.41	1.091
Carbohydrates	L-Arabinose	0.416	0.913
Carbohydrates	D-Glucuronic acid	0.419	0.954
Alkylamines	Ratio of Ethanolamine/O-Phosphoethanolamine	0.423	0.731
Amino Acid	D-2-Hydroxyglutaric acid	0.433	0.884
Carbohydrates	Ratio of D-Glucose/Sucrose	0.434	0.591
Organic Acids	Phosphoenolpyruvic acid	0.441	0.547
Organic Acids	Isocitric acid	0.443	0.873
Organic Acids	Maleic acid	0.458	0.813
Fatty Acids	Arachidic acid	0.459	0.867
Carbohydrates	Ratio of D-Glucuronic acid/Myoinositol	0.465	1.064
Amino Acid	Ratio of Ketoleucine/L-Leucine	0.471	1.249
Lipids	MG160	0.473	1.398
Indoles	Melatonin	0.476	0.833
Amino Acid	Ornithine	0.477	1.061
Organic Acids	Ratio of Fumaric acid/Succinic acid	0.479	0.407
Organic Acids	Ratio of Oxoglutaric acid/Isocitric acid	0.482	1.241
Amino Acid	Methionine sulfoxide	0.483	0.927
Nucleotide	Xanthine	0.483	1.323
Organic Acids	Picolinic acid	0.484	1.064
Organic Acids	Oxoglutaric acid	0.484	1.353
Nucleotide	Uracil	0.493	0.657
Alkylamines	Putrescine	0.495	1.094
Fatty Acids	Caproic acid	0.5	0.907
Organic Acids	Azelaic acid	0.506	1.074
Organic Acids	Vanillylmandelic acid	0.527	1.093
Fatty Acids	Oleic acid	0.53	3.269
Carbohydrates	D-Glucose	0.535	1.58
Organic Acids	4-Hydroxyphenylpyruvic acid	0.539	0.761
aldehydes	Glyceraldehyde	0.543	0.974
Nucleotide	Ratio of Uracil/Uridine	0.543	0.853
Lipids	Hexanoylcarnitine	0.549	0.994
Organic Acids	Fumaric acid	0.562	0.958
Carbohydrates	Rhamnose	0.566	1.008
Nucleotide	Uridine	0.567	1.112
Amino Acid	L-Isoleucine	0.574	1.318
Organic Acids	Citric acid	0.584	1.011
Amino Acid	Ratio of Citrulline/L-Arginine	0.584	0.981
Amino Acid	Beta-Alanine	0.598	1.048
Hormone	Normetanephrine	0.603	1.185
Carbohydrates	D-Fructose	0.607	2.627
Amino Acid	Aminoadipic acid	0.608	0.935
Nucleotide	Allantoin	0.62	1.031

Organic Acids	Methylmalonic acid	0.625	5.534
Carbohydrates	Erythrose	0.633	1.045
Alkylamines	Ratio of Spermidine/Putrescine	0.634	0.891
Carbohydrates	Ratio of L-Arabinose/L-Arabitol	0.636	0.908
Fatty Acids	Heptadecanoic acid	0.636	1.02
Vitamin	Pyridoxine	0.636	0.799
Organic Acids	Oxalic acid	0.639	0.889
Carbohydrates	D-Ribose	0.642	1.064
Carbohydrates	Sorbitol	0.644	0.575
Amino Acid	L-Phenylalanine	0.654	0.929
Vitamin	Pantothenic acid	0.664	4.647
Organic Acids	Hydroxypropionic acid	0.667	1.184
Organic Acids	Hippuric acid	0.67	0.924
Phenols	5-Hydroxydopamine	0.678	0.975
Carbohydrates	Galactonic acid	0.713	1.085
Organic Acids	Uric acid	0.722	0.936
Amino Acid	L-Norleucine	0.736	0.948
Amino Acid	3-Oxoalanine	0.739	1.014
Amino Acid	L-Homoserine	0.739	1.096
Alcohols	Glycerol	0.746	0.998
Fatty Acids	Docosahexaenoic acid	0.746	1.177
Organic Acids	L-Pipecolic acid	0.747	3.16
Amino Acid	Ratio of Sarcosine/Glycine	0.751	1.669
Phenols	Dopamine	0.771	1.405
Organic Acids	Glyceric acid	0.772	0.928
Carbohydrates	D-Galactose	0.785	0.927
Organic Acids	Taurine	0.787	0.668
Lipids	O-Phosphoethanolamine	0.806	1.068
Alcohols	Myoinositol	0.824	0.965
Amino Acid	Sarcosine	0.827	1.074
Fatty Acids	Dodecanoic acid	0.83	0.995
Organic Acids	3-Pyridylacetic acid	0.834	1.052
Fatty Acids	Pelargonic acid	0.834	1.011
Organic Acids	Pyrophosphate	0.835	1.303
Amino Acid	Ratio of L-Serine/Glycine	0.842	1.117
Fatty Acids	Nonadecanoic acid	0.844	1.015
Alkylamines	Ethanolamine	0.865	0.928
Nucleotide	Ratio of Xanthine/Hypoxanthine	0.869	0.812
Organic Acids	Nicotinic acid	0.88	1.074
Alcohols	2-Hydroxypyridine	0.897	1.002
Carbohydrates	Mannitol	0.899	0.928
Fatty Acids	Palmitic acid	0.905	0.964
Fatty Acids	Stearic acid	0.917	1.004
Amino Acid	Glycine	0.92	1.082
Carbohydrates	D-2,3-Dihydroxypropanoic acid	0.933	1.185
Organic Acids	4-Hydroxycinnamic acid	0.935	1.232
Organic Acids	Adipic acid	0.941	0.994
Amino Acid	L-Serine	0.943	0.975
Organic Acids	Succinic acid	0.965	3.25
Nucleotide	Hypoxanthine	0.967	1.493
Amino Acid	Ratio of Glycine/L-Serine	0.97	0.895

Fatty Acids	Linoleic acid	0.976	1.192
Fatty Acids	Myristic acid	0.995	0.915
