# Supplementary material for: Metabolomic Study on the Therapeutic Effect of the Jianpi Yangzheng Xiaozheng Decoction on Gastric Cancer Treated with Chemotherapy Based on GC-TOFMS Analysis
Source: Evid Based Complement Alternat Med. 2021 Mar 17;2021:8832996. doi: 10.1155/2021/8832996 (PMC7994103; doi:10.1155/2021/8832996)
Supplement: Supplementary Materials — Table S1: different metabolites via multivariate statistical analysis (VIP > 1, group C vs group A); Table S2: different metabolites via univariate statistical analysis (group C vs group A); Table S3: different metabolites via univariate statistical analysis (group B vs group A). [file 8832996.f1.zip › 8832996.f1/Supplementary material 3.pdf]

Class	Name	Pvalue	FC
Amino Acid	Ratio of Citrulline/L-Arginine	1.10E-03	0.786
Amino Acid	L-Homoserine	3.00E-03	1.853
Hormone	Normetanephrine	1.00E-02	2.038
Fatty Acids	Behenic acid	1.50E-02	0.582
Amino Acid	L-Alloisoleucine	2.10E-02	1.5
Amino Acid	Ratio of L-Serine/Glycine	2.10E-02	2.374
Amino Acid	Ratio of Glycine/L-Serine	2.10E-02	0.426
Amino Acid	Ratio of Citrulline/Ornithine	2.10E-02	0.76
Organic Acids	3-Methyl-2-oxovaleric acid	2.40E-02	0.83
Lipids	Decanoylcarnitine	2.80E-02	0.534
Amino Acid	Glycine	3.80E-02	0.656
Carbohydrates	Gluconolactone	3.80E-02	0.761
Amino Acid	Ratio of Ketoleucine/L-Leucine	5.00E-02	0.736
Organic Acids	Benzoic acid	0.065	0.741
Carbohydrates	D-2,3-Dihydroxypropanoic acid	0.065	3.655
Carbohydrates	L-Sorbose	0.065	0.831
Fatty Acids	Stearic acid	0.065	0.837
Amino Acid	Ratio of Sarcosine/Glycine	0.065	5.131
Amino Acid	L-Leucine	0.083	1.439
Amino Acid	Ornithine	0.083	1.412
Amino Acid	Dimethylglycine	0.105	1.797
Amino Acid	L-Norleucine	0.105	0.72
Carbohydrates	D-Tagatose	0.105	0.93
Alkylamines	Ratio of Spermidine/Putrescine	0.105	0.601
Alcohols	2-Hydroxypyridine	0.13	1.076
Alkylamines	Putrescine	0.13	2.102
Amino Acid	L-Histidine	0.13	1.122
Carbohydrates	D-Glucose	0.13	0.606
Nucleotide	Ratio of Hypoxanthine/Inosine	0.13	1.911
Amino Acid	Ratio of L-Asparagine/L-Aspartic acid	0.13	1.388
Carbohydrates	Mannitol	0.161	0.556
Fatty Acids	Arachidic acid	0.161	0.799
Lipids	Cholesterol	0.161	0.856
Organic Acids	Vanillylmandelic acid	0.189	0.787
Amino Acid	L-Proline	0.195	1.302
Organic Acids	Pimelic acid	0.195	0.727
Organic Acids	Taurine	0.195	0.3
Carbohydrates	D-Glucuronic acid	0.195	1.138
Indoles	Ratio of Tryptamine/L-Tryptophan	0.195	0.783
Organic Acids	L-Pipecolic acid	0.234	3.736
Organic Acids	Glutaric acid	0.234	0.941
Amino Acid	L-Asparagine	0.234	1.181
Nucleotide	Adenine	0.234	1.256
Indoles	Melatonin	0.234	0.471
Fatty Acids	Tetracosanoic acid	0.234	0.761
Organic Acids	3-Pyridylacetic acid	0.27	1.287
Alkylamines	Ethanolamine	0.279	0.956
Fatty Acids	Myristic acid	0.279	0.787
Carbohydrates	D-Mannose	0.279	1.233
Fatty Acids	Palmitoleic acid	0.279	0.763

Amino Acid	L-Cystine	0.279	1.307
Nucleotide	Uridine	0.279	0.854
Organic Acids	Picolinic acid	0.293	0.781
Amino Acid	L-Valine	0.328	1.222
Carbohydrates	Ribonolactone	0.328	0.733
Amino Acid	1-Methylhistidine	0.328	1.406
Fatty Acids	Palmitic acid	0.328	0.833
Fatty Acids	Arachidonic acid	0.328	0.809
Carbohydrates	Ratio of D-Glucuronic acid/Myoinositol	0.328	1.664
Organic Acids	Ratio of Fumaric acid/Succinic acid	0.328	0.367
Carbohydrates	D-Threitol	0.344	1.389
Organic Acids	Glycolic acid	0.382	0.883
Amino Acid	Ketoleucine	0.382	0.708
aldehydes	Glyceraldehyde	0.382	0.83
Nucleotide	Uracil	0.382	0.654
Fatty Acids	Pelargonic acid	0.382	0.979
Amino Acid	L-Arginine	0.382	1.155
Fatty Acids	Docosahexaenoic acid	0.382	0.609
Amino Acid	Ratio of Beta-Alanine/L-Aspartic acid	0.382	1.37
Lipids	Hexanoylcarnitine	0.442	0.785
Organic Acids	2-Hydroxy-3-methylbutyric acid	0.442	0.785
Amino Acid	L-Alpha-aminobutyric acid	0.442	0.736
Organic Acids	Succinic acid	0.442	2.195
Amino Acid	Methylcysteine	0.442	1.386
Organic Acids	Adipic acid	0.442	0.878
Amino Acid	Pyroglutamic acid	0.442	1.12
Carbohydrates	D-Ribose	0.442	1.584
Nucleotide	Hypoxanthine	0.442	1.334
Carbohydrates	1,5-Anhydrosorbitol	0.442	0.827
Carbohydrates	D-Fructose	0.442	3.601
Organic Acids	Uric acid	0.442	0.694
Fatty Acids	Linoleic acid	0.442	0.789
Organic Acids	Petroselinic acid	0.442	1.061
Carbohydrates	Ratio of Gluconic acid/Gluconolactone	0.442	1.298
Organic Acids	Ratio of Uric acid/Xanthine	0.442	0.575
Organic Acids	Malonic acid	0.505	0.931
Organic Acids	Nicotinic acid	0.505	0.738
Organic Acids	Maleic acid	0.505	1.344
Amino Acid	L-Serine	0.505	0.994
Amino Acid	L-Aspartic acid	0.505	0.81
Organic Acids	4-Hydroxyphenylpyruvic acid	0.505	1.482
Carbohydrates	Allose	0.505	1.49
Organic Acids	4-Hydroxycinnamic acid	0.505	1.24
Vitamin	Pantothenic acid	0.505	4.216
Amino Acid	L-Alanine	0.574	0.989
Amino Acid	Sarcosine	0.574	2.008
Organic Acids	Pyrophosphate	0.574	1.392
Amino Acid	L-Phenylalanine	0.574	1.019
Carbohydrates	Sorbitol	0.574	1.162
Nucleotide	Xanthine	0.574	1.268
Indoles	Tryptamine	0.574	0.938

Lipids	MG182	0.574	0.792
Amino Acid	Ratio of 4-Hydroxyproline/L-Proline	0.574	0.73
Amino Acid	Ratio of Ornithine/L-Arginine	0.574	1.053
Carbohydrates	Ratio of L-Arabinose/L-Arabitol	0.574	0.912
Organic Acids	Pyrrole-2-carboxylic acid	0.636	0.933
Organic Acids	L-Lactic acid	0.645	0.972
NA	3-hydroxypyridine	0.645	0.918
Amino Acid	Urea	0.645	0.998
Organic Acids	Fumaric acid	0.645	0.816
Amino Acid	3-Oxoalanine	0.645	1.228
Amino Acid	Citrulline	0.645	0.871
Organic Acids	Citric acid	0.645	1.268
Organic Acids	Isocitric acid	0.645	0.912
Carbohydrates	Galactonic acid	0.645	1.222
Alcohols	Myoinositol	0.645	0.908
Fatty Acids	Elaidic acid	0.645	1.137
Nucleotide	Inosine	0.645	0.745
Carbohydrates	Alpha-Lactose	0.645	0.773
Amino Acid	Ratio of 4-Hydroxyphenylpyruvic acid/L-Tyrosine	0.645	1.479
Carbohydrates	Ratio of D-Fructose/Sucrose	0.645	1.123
Phosphate	Phosphate	0.721	0.837
Organic Acids	Glyceric acid	0.721	1.657
Amino Acid	Acetylglycine	0.721	1.073
Amino Acid	Aminomalonic acid	0.721	1.075
Carbohydrates	Threonic acid	0.721	1.091
Organic Acids	4-Hydroxybenzoic acid	0.721	0.951
Amino Acid	Homocysteine	0.721	1.286
Amino Acid	Aminoadipic acid	0.721	0.793
Carbohydrates	L-Arabitol	0.721	1.122
Carbohydrates	Rhamnose	0.721	1.277
Amino Acid	L-Glutamine	0.721	1.141
Phenols	Dopamine	0.721	1.176
Fatty Acids	Heptadecanoic acid	0.721	0.97
Fatty Acids	Oleic acid	0.721	4.632
Carbohydrates	Ratio of D-Glucose/Sucrose	0.721	0.965
Alkylamines	Ratio of Ethanolamine/O-Phosphoethanolamine	0.721	0.796
Amino Acid	Ratio of L-Tyrosine/L-Phenylalanine	0.721	0.965
Nucleotide	Ratio of Uracil/Uridine	0.721	0.826
Organic Acids	Methylmalonic acid	0.798	0.977
Amino Acid	Beta-Alanine	0.798	0.937
Organic Acids	Malic acid	0.798	1.188
Carbohydrates	Erythritol	0.798	1.042
Amino Acid	Creatine	0.798	1.555
Organic Acids	Hydroxypropionic acid	0.798	0.973
Organic Acids	Oxoglutaric acid	0.798	1.066
Carbohydrates	D-Xylose	0.798	1.031
Amino Acid	Methionine sulfoxide	0.798	1.021
Carbohydrates	D-Galactose	0.798	0.991
Amino Acid	L-Tyrosine	0.798	0.785
Phenols	5-Hydroxydopamine	0.798	0.911
Alkylamines	Spermidine	0.798	0.961

Lipids	Ratio of Glycerol 3-phosphate/Glycerol	0.798	1.048
Nucleotide	Ratio of Xanthine/Hypoxanthine	0.798	0.88
Amino Acid	Ratio of Pyruvic acid/L-Serine	0.798	1.228
Organic Acids	Ratio of Oxoglutaric acid/Isocitric acid	0.798	0.756
Organic Acids	Pyruvic acid	0.878	0.949
Amino Acid	Alpha-ketoisovaleric acid	0.878	0.901
Organic Acids	Oxalic acid	0.878	0.931
Alcohols	Glycerol	0.878	0.869
Amino Acid	4-Hydroxyproline	0.878	1.303
Amino Acid	Creatinine	0.878	0.946
Amino Acid	D-2-Hydroxyglutaric acid	0.878	1.051
Fatty Acids	Dodecanoic acid	0.878	0.96
Organic Acids	Azelaic acid	0.878	0.86
Lipids	O-Phosphoethanolamine	0.878	0.986
Organic Acids	Hippuric acid	0.878	0.779
Amino Acid	Ratio of L-Valine/Alpha-ketoisovaleric acid	0.878	0.959
Amino Acid	Ratio of Alpha-ketoisovaleric acid/L-Valine	0.878	1.045
Amino Acid	Ratio of L-Glutamine/L-Glutamic acid	0.878	1.497
Amino Acid	Ratio of L-Glutamic acid/L-Glutamine	0.878	0.693
Organic Acids	Ratio of Pyruvic acid/L-Lactic acid	0.878	0.859
Amino Acid	Ratio of Urea/L-Arginine	0.878	0.889
Fatty Acids	Nonadecanoic acid	0.916	0.964
Amino Acid	L-Isoleucine	0.959	1
Amino Acid	L-Threonine	0.959	1.042
Amino Acid	L-Cysteine	0.959	1.123
Amino Acid	L-Lysine	0.959	0.992
Indoles	3-Indolepropionic acid	0.959	1.052
Amino Acid	L-Tryptophan	0.959	0.923
Carbohydrates	D-Maltose	0.959	1.233
Fatty Acids	Caproic acid	0.959	0.979
Alkylamines	Hydroxylamine	0.959	0.939
Amino Acid	L-Methionine	0.959	1.074
Organic Acids	Phosphoenolpyruvic acid	0.959	0.723
Amino Acid	L-Glutamic acid	0.959	0.88
Carbohydrates	L-Arabinose	0.959	1.213
Carbohydrates	Ribitol	0.959	1.057
Lipids	Glycerol 3-phosphate	0.959	1.063
Carbohydrates	Gluconic acid	0.959	1.016
Lipids	MG160	0.959	0.884
Vitamin	Alpha-Tocopherol	0.959	0.916
Amino Acid	Ratio of L-Glutamic acid/Pyroglutamic acid	0.959	0.706
Amino Acid	Ratio of Putrescine/Ornithine	0.959	1.1
Organic Acids	Ratio of Pyruvic acid/Phosphoenolpyruvic acid	0.959	1.612
Amino Acid	2-Hydroxybutyric acid	1	1.08
Carbohydrates	Erythrose	1	1.043
Nucleotide	Allantoin	1	0.964
Vitamin	Pyridoxine	1	0.937
Carbohydrates	Sucrose	1	1.306
Lipids	MG181	1	0.948
Amino Acid	Ratio of Sarcosine/Dimethylglycine	1	0.986
Amino Acid	Ratio of Oxoglutaric acid/L-Glutamic acid	1	1.006

Amino Acid	Ratio of L-Glutamic acid/Oxoglutaric acid	1	1.031
Amino Acid	Ratio of Pyruvic acid/L-Alanine	1	0.995
